# Machine Learning Classification of Local Environments in Molecular Crystals

**DOI:** 10.1021/acs.jctc.4c00418

**Published:** 2024-07-03

**Authors:** Daisuke Kuroshima, Michael Kilgour, Mark E. Tuckerman, Jutta Rogal

**Affiliations:** † Department of Chemistry, New York University (NYU), New York, New York 10003, United States; ‡ Courant Institute of Mathematical Sciences, New York University, New York, New York 10012, United States; § NYU-ECNU Center for Computational Chemistry at NYU Shanghai, 3663 Zhongshan Rd. North, Shanghai 200062, China; ∥ Simons Center for Computational Physical Chemistry at New York University, New York, New York 10003, United States; ⊥ Fachbereich Physik, Freie Universität Berlin, Berlin 14195, Germany

## Abstract

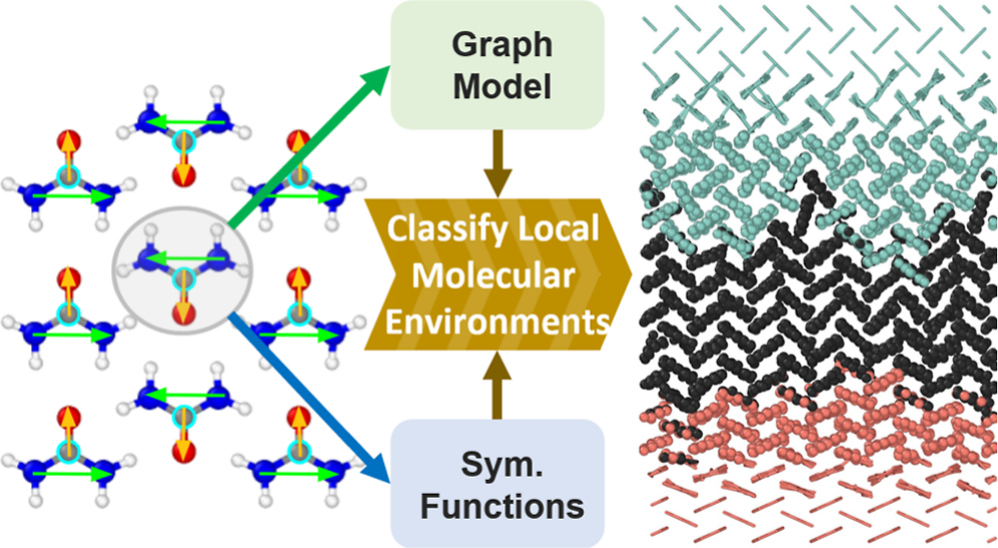

Identifying local structural motifs and packing patterns of molecular solids is a challenging task for both simulation and experiment. We demonstrate two novel approaches to characterize local environments in different polymorphs of molecular crystals using learning models that employ either flexibly learned or handcrafted molecular representations. In the first case, we follow our earlier work on graph learning in molecular crystals, deploying an atomistic graph convolutional network combined with molecule-wise aggregation to enable per-molecule environmental classification. For the second model, we develop a new set of descriptors based on symmetry functions combined with a point-vector representation of the molecules, encoding information about the positions and relative orientations of the molecule. We demonstrate very high classification accuracy for both approaches on urea and nicotinamide crystal polymorphs and practical applications to the analysis of dynamical trajectory data for nanocrystals and solid–solid interfaces. Both architectures are applicable to a wide range of molecules and diverse topologies, providing an essential step in the exploration of complex condensed matter phenomena.

## 1 Introduction

Elucidation of the microscopic structure of molecular materials is key to predicting and engineering their properties. Despite significant advances in experimental techniques, following structural transformations in condensed-phase systems with atomistic resolution remains a challenge due to the time and length scales involved. Computational approaches, such as molecular dynamics (MD) simulations, have become an invaluable tool to provide such microscopic insights, but characterizing the structural features of a molecular system from the simulation data is, in general, nontrivial. However, following the dynamical evolution of local structural environments is essential when studying polymorphic transitions, especially concerning the complex atomistic processes that govern nucleation and growth.

A number of descriptors have been developed over the years to capture local or global structural features, including Steinhardt order parameters,^1,2^ common neighbor analysis,^3−5^ entropy-based fingerprints,^6^ smooth overlap of atomic positions,^7^ and atom-centered symmetry functions (SFs)^8^ (see also refs (9–16) for further overviews and examples). More recently, machine learning has been utilized to classify local environments with both supervised and unsupervised approaches.^17−28^ These machine learning models for local structure classification fall into two broad categories: models that use handcrafted structural features or descriptors together with a simple classification model, and models that use only very general information, such as atom types and distances, wherein the models learn the structural representation and intermolecular correlations simultaneously. The former approach is attractive in its ostensible simplicity but relies on the development of high-quality descriptors; the latter requires a more complex model architecture, is less intuitive concerning the system, but is more generally applicable. Here, graph neural network (GNN) approaches are attractive in their generality, allowing one to use a single flexible model for most systems. GNNs have also been used to describe condensed-phase systems, in which the relevant features are learned in a “ground up” fashion from basic atomistic information.^26,27,29−38^

The structure characterization methods discussed above have been primarily established in the context of atomistic condensed-matter systems. In molecular systems, additional challenges arise since not only the positions of the molecules but also their relative orientation as well as conformational changes need to be accounted for. One idea is to include this information via a point-vector representation of the molecules where, for example, the center of mass denotes the molecule position and vectors denote the absolute orientation of a given molecule, such that these two elements can be combined into suitable descriptors.^39,40^

In this work, we advance the state-of-the-art of machine learning classification of local environments to capture the complex structural features in molecular solids. We present two parallel approaches, one based on handcrafted descriptors and the other based on learned feature embeddings. The handcrafted descriptors extend our previous work on atomistic systems^19^ to molecular SFs by combining the SFs with a point-vector representation of the molecules. For the learned embeddings, we utilize our recently introduced molecular crystal graph model MXtalNet^41^ and augment the architecture with a classification task. The trained models are able to distinguish different local environments in various polymorphs of complex molecular solids with high accuracy. Furthermore, both approaches are applicable to a wide range of systems, including clusters and interfaces, and can provide time-resolved information regarding melting transitions or solid–solid transformations. The potential of our classification models is exemplified for urea and nicotinamide, but the methods are easily extended to arbitrary molecules. The approaches presented introduce an essential and valuable component in the analysis and interpretation of simulation data for molecular solids.

## 2 Model Architectures and Training

**Figure 1:**
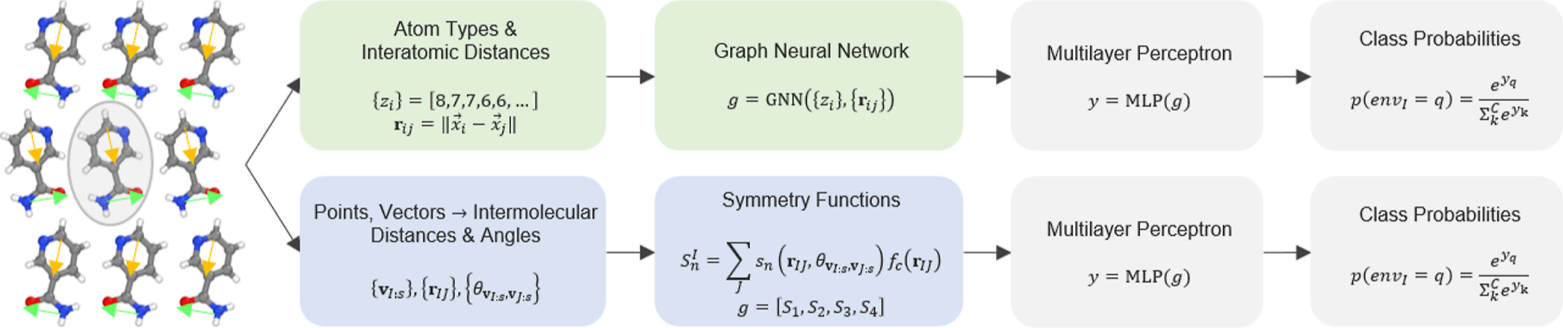
Workflow of the GNN and SF classifiers on top and bottom, respectively, including molecule representation, local embedding, and classification. The GNN learns the features *g* used in the classification task, while for the SF classifier, the features *g* are given by the handcrafted molecular SFs.

The general idea of our two model architectures is schematically illustrated in Figure 1. The classification is performed for each molecule to characterize its local structural environment. An appropriate model should be invariant to permutations of atoms of the same types, as well as global translations, rotations, and inversions of the atomic coordinates, focusing only on the structural correlations which define the respective polymorphs. For the learned feature embedding, the positions and atom types of a given molecule and its neighbors comprise the input to a GNN coupled with a multilayer perceptron (MLP) to perform classification on the final embedding. For the handcrafted features, the atomic positions are used to construct a point-vector representation for each molecule, which is then employed to compute a set of molecular SFs as input to the classification MLP. Details of the model architectures and training protocols are given in the proceeding subsections.

### 2.1 Molecular Crystal Graph Network

For the molecular GNN, we used a relatively straightforward GNN, similar in geometric complexity to SchNet,^42^ taking interatomic distances and atom types as inputs. This GNN encodes pairwise interatomic distances to edge embeddings, atom types to node embeddings, and performs graph convolutions via the TransformerConv operator^43^ implemented in the Torch Geometric package.^44^

The GNN parses a single sample in the following way, starting with the embedding of the input nodes atom types *z*_*i*_
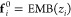
1with EMB as a learnable discrete embedding function, followed by a fully connected layer. The edge embedding is
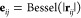
2where |**r**_*ij*_| is the distance between nodes *i* and *j*, and Bessel is the radial embedding function from DimeNet^45^ with cutoff *r*_c_ = 6 Å and a basis of 32 spherical Bessel functions. A fully connected layer is defined as FC(**x**) = **W** · **x** + **b**, with **W** and **b** as learnable parameters. Messages are passed between nodes, conditioned on node and edge embeddings via eqs 3 for edge → message and (4) for node → message over *N* graph convolutions, with GC being the graph convolution operation
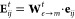
3
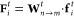
4
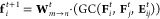
5

After each graph convolution, the node embeddings are passed through a fully connected layer with residual connection
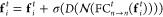
6with σ being the activation function (here GeLU^46^), *D*(*x*), a dropout function, and , the graph layer norm operation. The final node features, corresponding to information about each atom and its local environment, are aggregated into a single embedding vector representing the entire molecule and input to a two-layer activated fully connected network with layer normalization and dropout, followed by a reshaping to the number of possible classes. Although there are currently many powerful graph aggregators, we find that max aggregation, i.e., selecting the maximum value from each feature channel, *k*, across the final atomic node embeddings in each molecule, is simple and efficient for learning the desired functions with
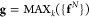
7and
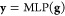
8with MLP being a multilayer perceptron. The class probabilities for a molecule *I* being in a particular environment *q* are computed via the softmax activation function
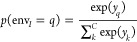
9with *C* being the number of possible environments.

We found that one or two graph convolutions gave similar performance, although more convolutions result in a larger volume for what the model considers as a “local environment”. The number of convolutions depends on the user’s desired sensitivity to longer-range structural correlations, but in the current examples, more than two convolutions resulted in training instability and overall poor convergence. For other hyperparameters, optimal performance was obtained with a relatively deep embedding (256 for node and graph embeddings, 128 for messages), aggressively regularized with layer norm and a dropout of 0.25 in graph convolutions, nodewise fully connected layers, and the embedding-to-output network. With these settings, the model converged via the Adam optimizer to the test minimum very quickly, usually within a few tens of epochs. Smaller models could certainly be explored, although we generally found convergence properties to be poorer in that regime. For further details of model construction, see the Supporting Information and our accompanying codebase.^47^

### 2.2 Molecular Symmetry Functions

**Figure 2:**
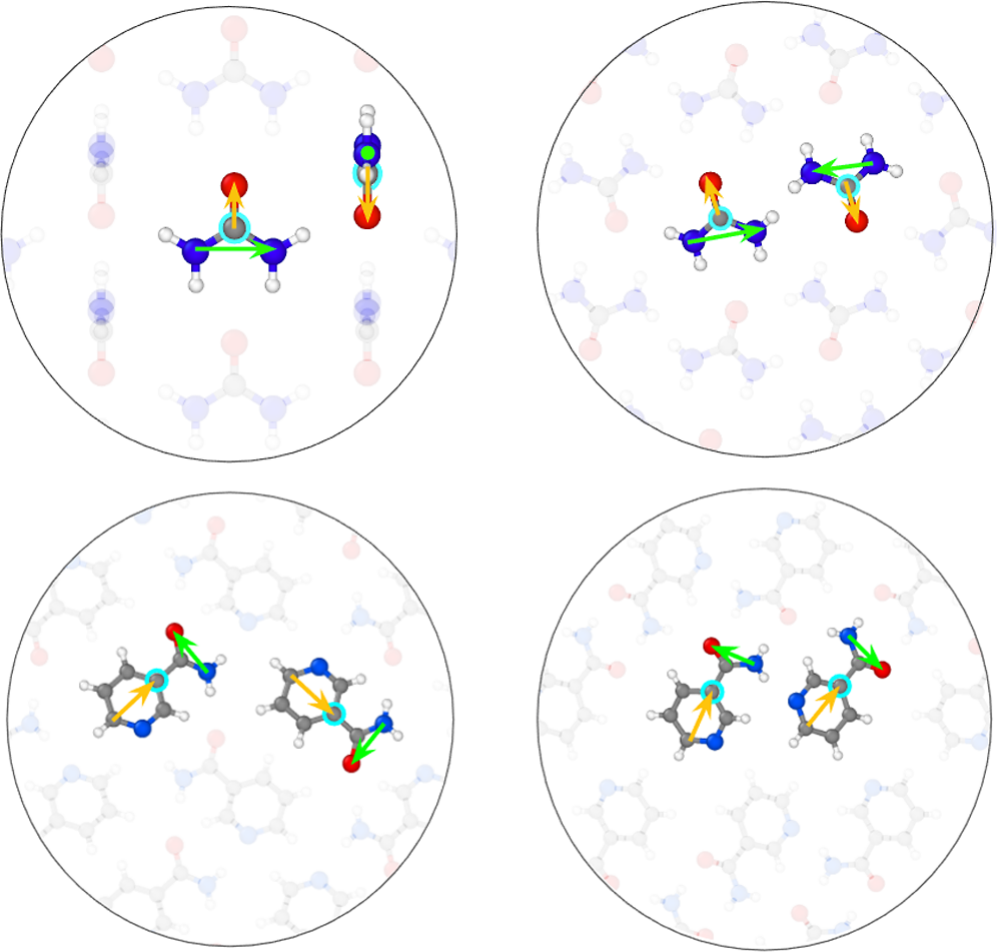
Point-vector representation for urea (top panels) and nicotinamide (bottom panels) in two different polymorphs, respectively. The turquoise circles indicate the positions of the molecules **r**_*I*_, and the green and orange vectors, **v**_*I*;1_ and **v**_*I*;2_, characterize their relative orientations.

Our second model derives a set of descriptors for each molecule based on the Behler–Parrinello SFs^8^ in combination with a point-vector representation^39,40^ of the molecules. The point-vector representations for urea and nicotinamide are illustrated in Figure 2, where the position **r**_*I*_ of molecule *I* is represented by a selected atom (indicated by a turquoise circle in Figure 2). Vectors **v**_*I*;*s*_ are defined between two selected atoms in the molecule, such that they can capture the relative orientations of the molecules (indicated in orange, **v**_*I*;1_, and green, **v**_*I*;2_, in Figure 2). We utilize four different types of molecular SFs *S*^*I*^. Two are akin to radial SFs for atomistic systems but using the molecule positions **r**_*I*_
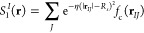
10and
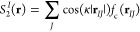
11where the sum runs over all other molecules, **r**_*IJ*_ = **r**_*J*_ – **r**_*I*_, *f*_c_ is a cutoff function (see Supporting Information for details), and η, *R*_*s*_, and κ are tunable parameters. The other two types of molecular SFs use the molecule vectors to characterize the relative orientation of molecule *I* with respect to its neighbors *J*
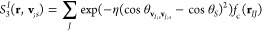
12and
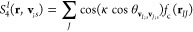
13where  is the angle between vectors **v**_;*s*_ on molecules *I* and *J*, and cos θ_*S*_ is another tunable parameter. The total number of employed molecular SFs is 24 for both urea and nicotinamide. Details of the selected molecular SFs and corresponding values of the tunable parameters are given in Tables S1 and S2 of the Supporting Information.

To perform classification of molecule environments, the molecular SF descriptors are input to a rather small MLP with two hidden layers, 25 nodes each, and the softmax activation in eq 9 for the output layer. A larger MLP with more hidden layers and nodes would provide greater flexibility, but due to the simplicity of the classification task, a small network was sufficient for our applications, making both the training and evaluation rather fast. For further implementation details, see the SF classifier codebase.^48^

### 2.3 Training the Models

Training data were generated by MD simulations of all crystal polymorphs and the melt for urea and nicotinamide. Simulations were performed using the lammps MD package^49^ with a general Amber force field (GAFF).^50^ Here, we briefly summarize the protocol for training the classification models. Further details regarding the MD simulations and training are given in the Supporting Information.

The graph classifier was trained on a mix of trajectory snapshots of periodic bulk cells approximately 20 × 20 × 20 Å^3^ and gas-phase spherical clusters with a diameter of ∼30 Å to give the effect of a “surface”. Molecules are identified as being on the surface if their local coordination number, CN_*I*_, is smaller than 20, with CN_*I*_ = ∑_*J*_θ[−(|**r**_*IJ*_| – *R*_c_)], where θ is the Heaviside function and *R*_c_ is the molecule radius plus the graph convolution cutoff. Due to differences in the architecture of the classification MLP, the SF classifier was trained on periodic bulk samples alone.

We train the classification models on stable, low-temperature snapshots of the known bulk polymorphs of each molecular crystal, as well as on the supercooled melt state. We test the models’ generalization performance on configurations from higher-temperature MD simulations, with adaptation to thermal noise standing in as a proxy for overall generalization. The specific temperatures for each of the studied systems are discussed together with the results below.

The graph classifier was trained until the test loss began to increase, and the model checkpoint at the test loss minimum was used for evaluation. Repeated retraining over several random seeds found variations in the test loss minimum of only a few percent between runs. We used a combined cross-entropy loss including both the loss for the local polymorph classification for each molecule and the molecule topology, that is, “surface” vs “bulk”.

The SF classifier was trained until the training loss converged, which, generally, resulted in very small test losses.

## 3 Classification of Local Environments

### 3.1 Bulk Polymorphs of Urea and Nicotinamide

**Figure 3:**
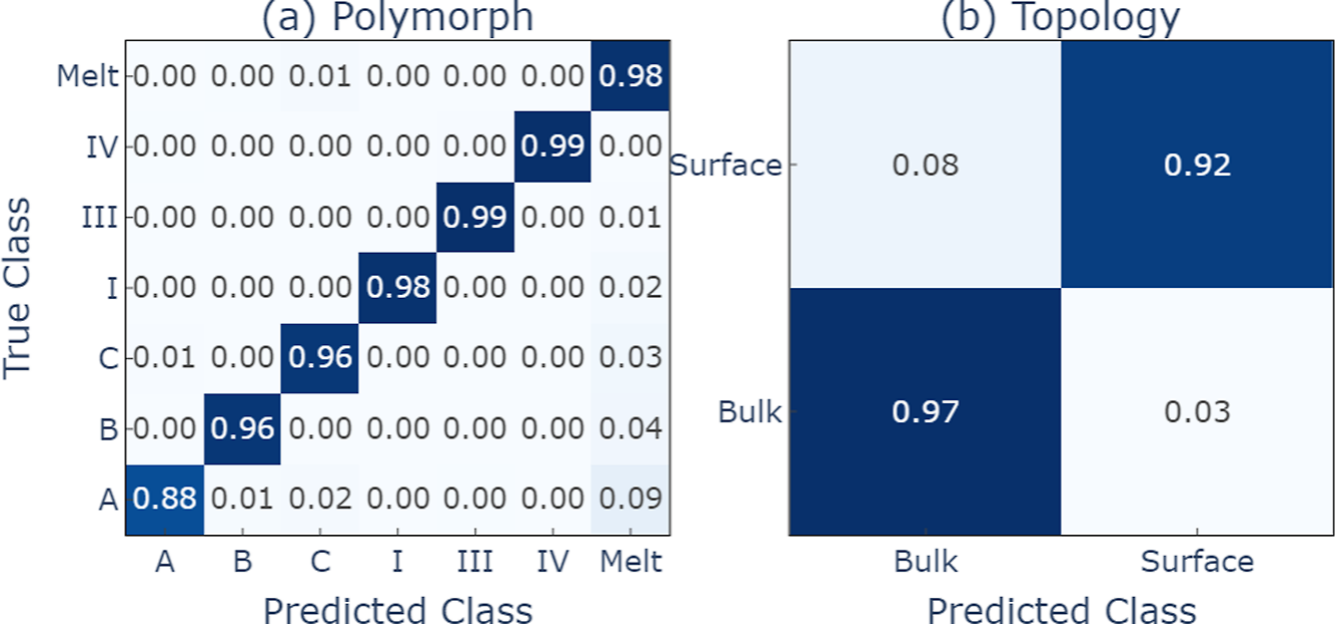
Confusion matrices for the graph classifier on the (a) polymorphs and (b) topologies of urea at 200 K for crystals and 350 K for the melt. Micro *F*1 scores = 0.969, 0.960.

We initially trained and applied our classification models to two different systems, urea and nicotinamide. Urea is a relatively small and rigid molecule which is also significantly polymorphic, having six distinct crystal structures with a unique intermolecular packing character^51−54^ (see Figure S1 of the Supporting Information for a visualization of the respective polymorphs). The models were trained on *T* = 100 K crystal samples and *T* = 350 K melts, and evaluation metrics were computed on samples at 200 K for the crystal polymorphs and 350 K for the melt. At low temperatures, the graph classifier achieves perfect accuracy for both polymorphs and local topologies. This means that the GNN learns an embedding where the different molecule environments are clearly separated without overlap. This is expected as the graph model is rather expressive, and in all the thousands of individual molecular environments, the local structure seen by the model should be quite similar within each polymorph. The graph model also generalizes well to higher temperature samples at *T* = 200 K, as evidenced by the confusion matrices shown in Figure 3, meaning that larger thermal fluctuations can be captured within the trained model. Only urea form A shows a slightly larger classification error, with about 9% of the samples being identified as “melt”, which might be due to the lower stability of form A. The SF classifier also demonstrated excellent performance on urea, achieving comparable or better performance at polymorph classification (*F*1 ≳ 0.98) to the GNN model in training and evaluation while being lightweight and fast to run at inference. The corresponding confusion matrix can be found in Figure S3 of the Supporting Information.

**Figure 4:**
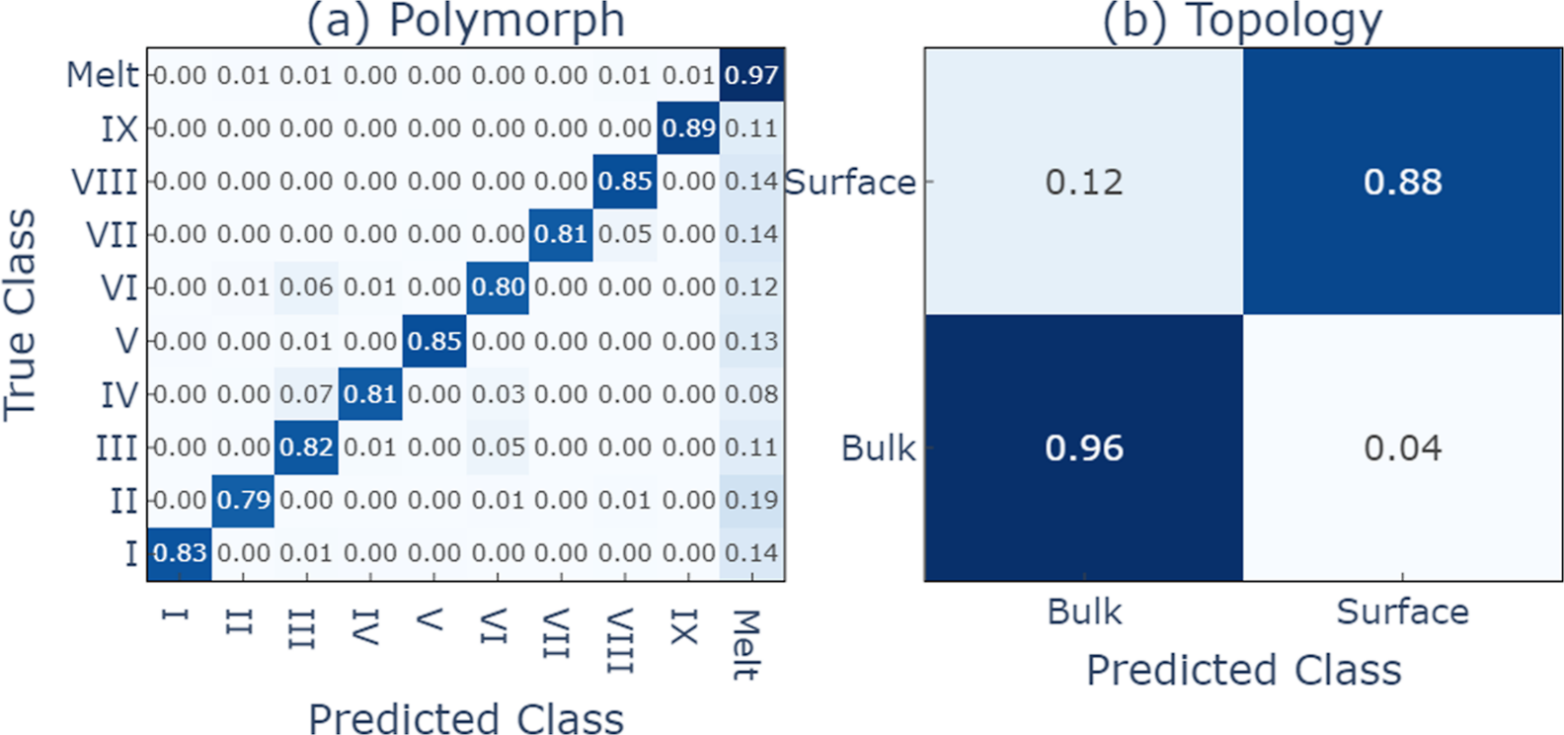
Confusion matrices for the graph classifier on the (a) polymorphs and (b) topologies of nicotinamide at 350 K. Micro *F*1 scores = 0.875, 0.922.

As a second example, we chose nicotinamide as a more challenging molecule. Nicotinamide is larger than urea and more flexible with internal degrees of freedom that allow for polymorphs consisting of different conformers of the molecule. Nine polymorphs of nicotinamide have been experimentally crystallized^55,56^ (see Figure S2 of the Supporting Information for a visualization of the respective polymorphs). Despite this significant added complexity in the molecular system, the performance of our classification models is again very good. As with urea, the training samples, both crystal polymorphs at 100 K and supercooled melts at 350 K, are essentially perfectly learned. The model also generalizes well to the high-temperature test samples of crystal polymorphs at 350 K. The corresponding confusion matrix for the GNN classifier is shown in Figure 4. The *F*1 score for nicotinamide at high temperatures is slightly worse than that for urea, 0.875 compared to 0.969, which reflects the increased flexibility in the thermal fluctuations at this even higher temperature. This is, however, not a fundamental limitation of the model as, when retrained with samples at both 100 and 350 K, the accuracy again approaches 100%.

We see that the generality and high capacity of the GNN model allow it to classify each polymorph and local topology without the need for model customization of any kind. Likewise, the SF classifier performs excellently on the nicotinamide polymorphs (see Figure S4 of the Supporting Information for the corresponding confusion matrices). This indicates that the chosen set of molecular SFs provides suitable descriptors to capture the additional complexity and flexibility in nicotinamide crystal polymorphs and melts.

One interesting point is that the GNN classifier exhibits somewhat lower performance on the nicotinamide high-temperature samples compared to the SF classifier when both are trained on low-temperature crystals and high-temperature melts alone. From the confusion matrices in Figure 4, it becomes clear that the accuracy loss of the graph classifier is primarily due to overprediction of the melt state. For a model trained only at 100 K and evaluated at 350 K, this should perhaps not be surprising. The larger thermal fluctuations in inter- and intramolecular degrees of freedom increase the general similarity of bulk crystals to the melt, and they are interpreted as such by the model. The fact that we do not see this effect as strongly in the SF classifier results indicates that the handcrafted descriptors are quite robust to fluctuations yet sensitive enough to achieve high classification accuracy.

**Figure 5:**
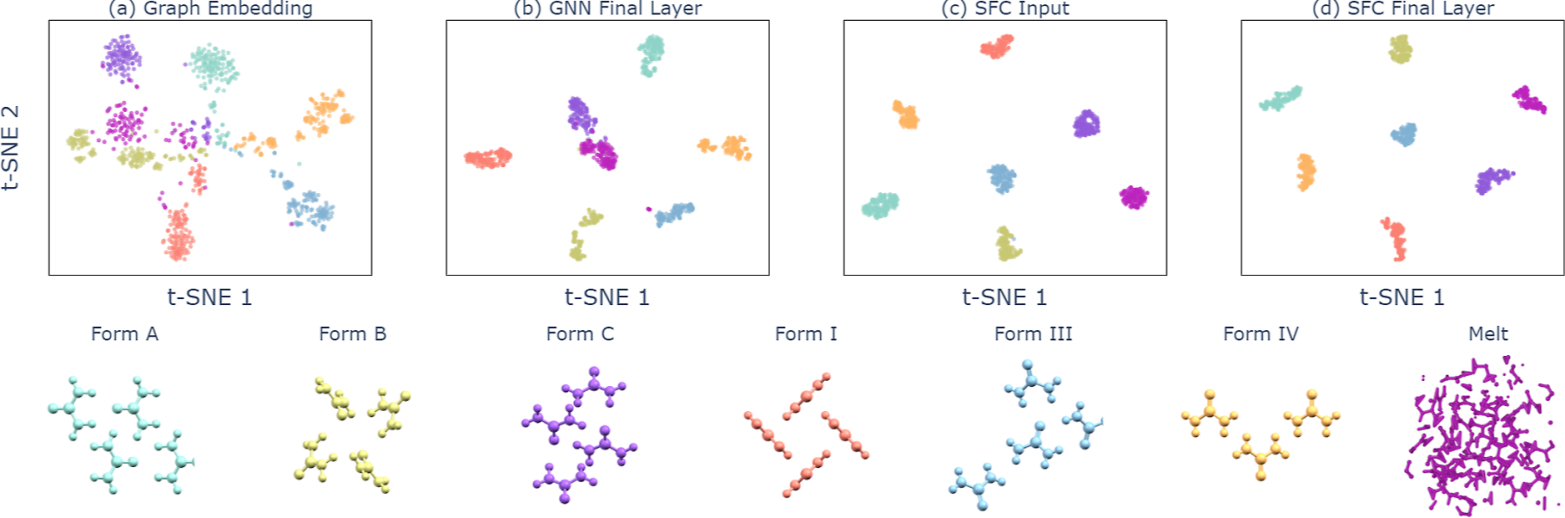
*T*-distributed stochastic neighbor embedding (*t*-SNE) of urea samples from the (a) 256-dimensional graph embedding (output of eq 7), (b) 256-dimensional final layer activation, (c) 24 SFs, and (d) 25-dimensional SFC final layer activation; samples are taken from three different temperatures of 100, 200, and 350 K.

To gain a better understanding of the learned and handcrafted features in our molecular graph and SF classifiers, respectively, we compare the corresponding embedding spaces. In Figure 5, the embedding spaces of the representations and final layer activations for urea are visualized using the *t*-distributed stochastic neighbor embedding (*t*-SNE).^57^Figure 5a shows that the molecular representation learned by the GNN already separates the different polymorphs of urea reasonably well. The quality of the handcrafted SFs is obvious when examining the *t*-SNE of the SF inputs in Figure 5c, which cluster essentially perfectly before applying any learned transformations. Figure 5b,d show the *t*-SNE of the final layer activations for the GNN and SF classifier, respectively. The class separation is excellent, as expected from the very high classification accuracy observed for both models.

**Figure 6:**
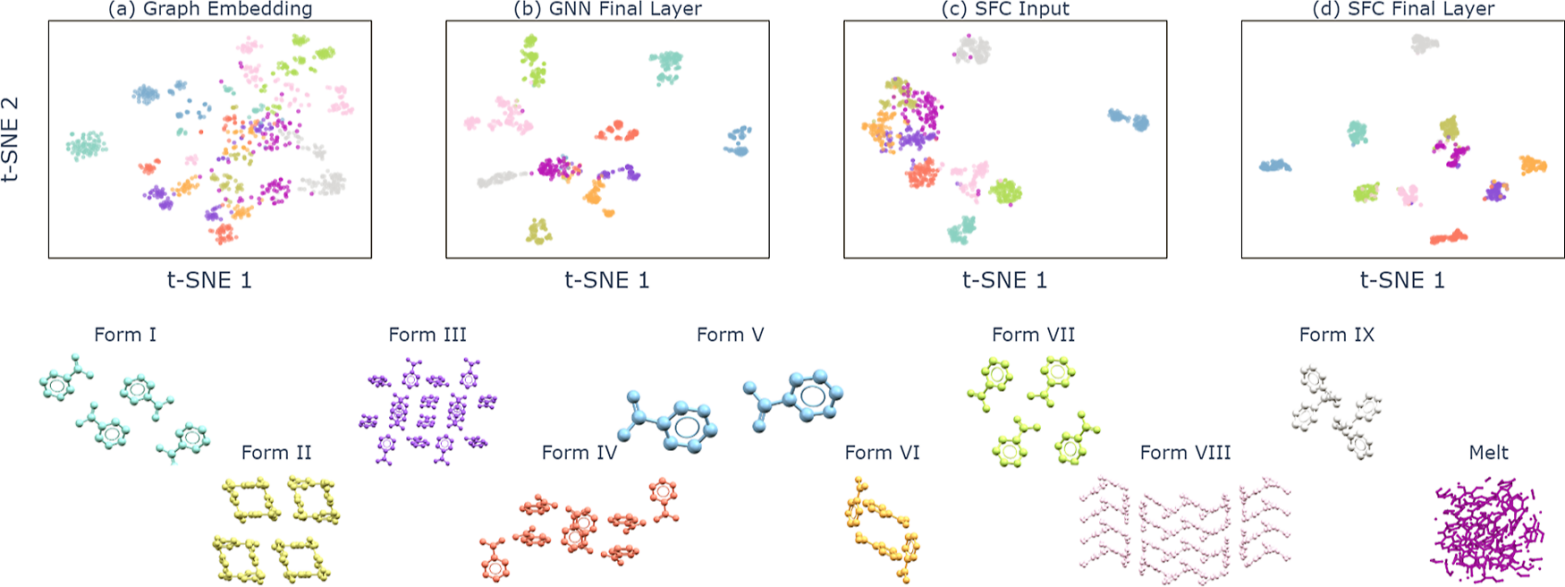
*t*-SNE of nicotinamide samples from the (a) graph embedding (output of eq 7), (b) final layer activation, (c) SFs, and (d) SFC final layer activation at temperatures of 100 and 350 K. Embedding dimensionality is the same as in Figure 5.

The *t*-SNE visualization of the embedding spaces for nicotinamide is shown in Figure 6. Both the learned and handcrafted embedding spaces in Figure 6a,c show imperfect classwise separation between the various polymorphs in nicotinamide. This again underscores the increased challenge in characterizing structural environments in more complex and flexible systems. In particular, samples from the melt seem to cover a wide range and are less clustered in the embedding spaces. We also see greater separation of samples from the same crystalline polymorphs in Figure 6a,b, including bifurcation of some classes, corresponding to the different sampled temperatures and topologies. The overlap between the melt and crystal embeddings visible in Figure 6a,b is also consistent with the GNN classifier confusing some crystalline polymorphs mainly with the melt, as seen in Figure 4. Nevertheless, the final learned representations in Figure 6b,d show again a very good separation between the different polymorph classes, even for the high-temperature samples.

### 3.2 Analyzing Molecular Simulations

An ability to characterize local environments reliably in unknown structures will be particularly useful when analyzing and interpreting trajectory data from molecular simulations. In the following, we discuss two examples: the stability of gas-phase nanocrystals at different temperatures and the migration of an interface during a solid–solid transformation in a molecular crystal.

#### 3.2.1 Dynamical Structure Characterization of Molecular Clusters

**Figure 7:**
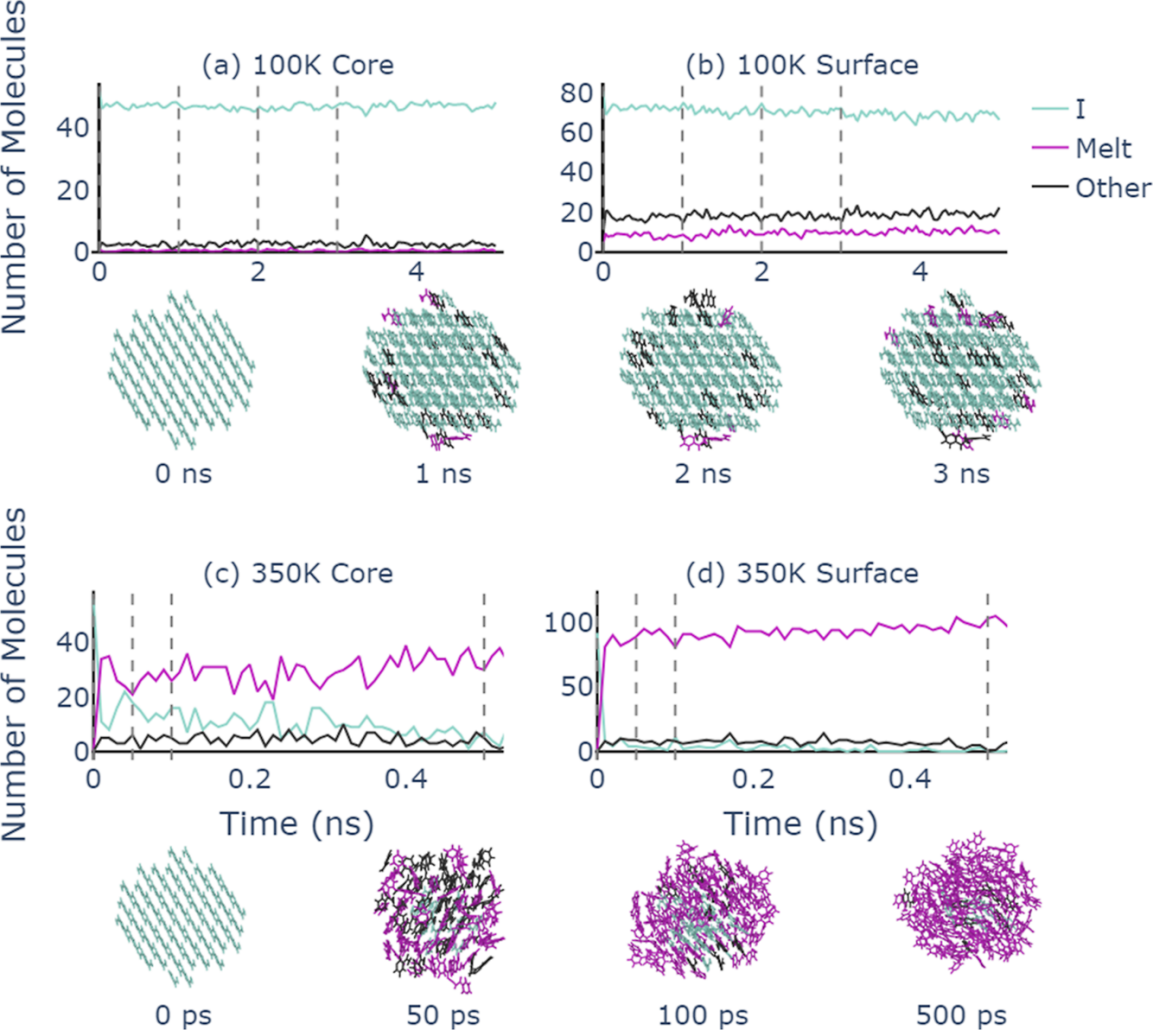
Time evolution of the number of molecules classified as form I, melt, or other (a,b) at 100 and (c,d) at 350 K. The analysis is shown separately for high-coordination “core” molecules in (a) and (c) and low-coordination “surface” molecules in (b) and (d). Vertical dashed lines identify the time points for the cluster snapshots, with molecules colored according to their most probable form. Snapshots were visualized using ovito.^58^

The GNN classifier trained on the bulk polymorphs of nicotinamide is used to identify the local environments of nicotinamide molecules in small nanocrystals. We set up spherical clusters of nicotinamide form I with a diameter of 34 Å containing 148 molecules. MD simulations for the clusters in vacuum are run at *T* = 100 and 350 K (further simulation details are given in the Supporting Information). In Figure 7, the structural evolution of the nicotinamide nanoclusters at these two temperatures is shown, obtained using the graph classifier.

Since the classifier provides information for each molecule individually, we can separate our analysis for molecules that are in the core region of the clusters, Figure 7a,c, and at their surfaces, Figure 7b,d. At 100 K, the nanocluster clearly keeps its crystalline structure over the entire simulation time. While the majority of molecules in the core region are identified as nicotinamide form I, molecules at the surface are partially classified as melt or others, which is expected since the structural environment at the surface is significantly different from the bulk. At 350 K, the crystalline cluster quickly melts starting from the surface. Within a few picoseconds, molecules at the surface are identified as liquid with a handful labeled as others. The core region melts a little more slowly with a few molecules initially remaining as form I and others. After approximately 500 ps, the cluster appears to be completely melted with only a small number of core molecules identified as others.

Despite not having been trained on clusters in vacuum or mixtures of polymorphs, the performance of our graph classifier in the analysis of the simulation data is sensible and very informative, allowing us to evaluate the structural stability and the onset of melting as a function of temperature.

#### 3.2.2 Time Evolution of Solid–Solid Phase Boundaries

Pushing our analysis tools even further, we apply our classification models to track the position of the interface between two different polymorphs of urea during a solid–solid transformation. A semicoherent interface between forms I and IV of urea is set up by pairing both phases along the [001] direction. The *xy*-dimensions parallel to the interface are fixed, resulting in 1.7% compression in *x* and 1.4% strain in *y* of urea I and 2.8% compression in *x* and 0.8% strain in *y* of urea IV, respectively. Periodic boundary conditions are applied in all dimensions, keeping molecules at one of the interfaces fixed, and simulations are run in the *NP*_*z*_*T* ensemble at *T* = 100 K (further simulation details are given in the Supporting Information).

**Figure 8:**
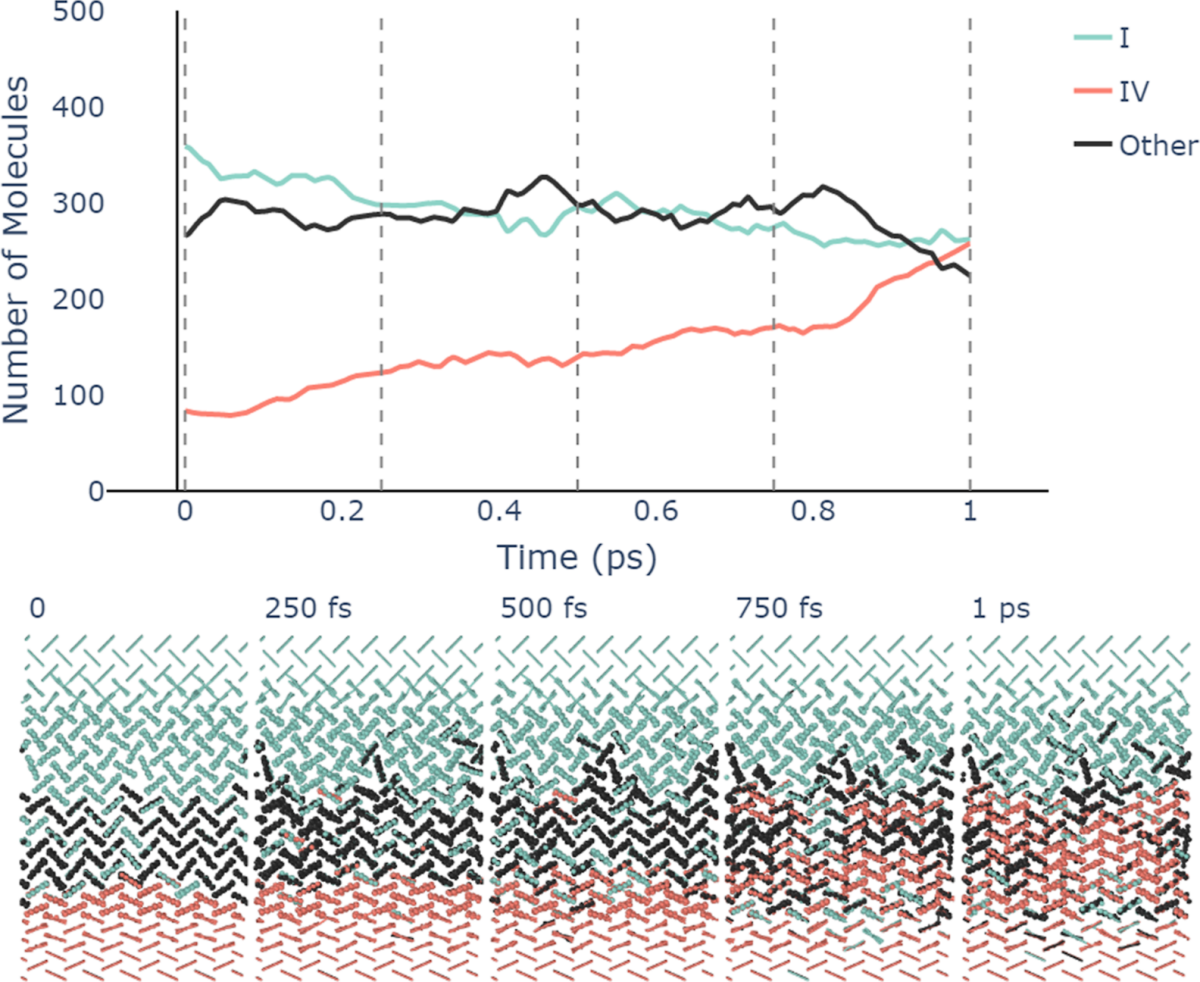
Time series of the molecule-wise composition of a system with a moving interface between forms I and IV of urea. In the top graph, only molecules in the central region of the simulation cell, highlighted in bold in the snapshots below, are included. Vertical dashed lines correspond to the time points from which the snapshots were sampled, with molecules colored according to their assigned polymorph. Snapshots were visualized using ovito.^58^

In Figure 8, the analysis of the structural transformation using the graph classifier is shown. Initially, the system is mainly composed of urea form I (green molecules) in the top half of the simulation cell with some form IV at the bottom. Molecules at the interface between the two polymorphs are primarily identified as “others” due to deviations in their local environments from the pure bulk polymorphs. Since within the chosen setup form I is rather unfavorable, transformation to form IV rapidly takes place over a few hundred femtoseconds, which is indicated by the continued increase of molecules identified as form IV and decrease of form I in the top graph of Figure 8.

Here, again, the utility of accurate local environment classification is clearly evidenced as subtle changes in local spacing and orientations of molecules can be seen to correspond to the transformation between distinct polymorphs, in this case forms I and IV of urea. Interestingly, we also see that the conversion from form I to IV is not perfect as some defects are left in the wake of the phase boundary as it moves upward through the sample.

## 4 Conclusions

We have introduced two machine learning-based approaches for the classification of local structural environments in molecular solids. Both the GNN classifier with learned feature embeddings and the SF classifier with handcrafted descriptors identify molecular environments in various bulk polymorphs with high accuracy. While the performance of the two machine learning models is comparable for the studied systems, there are differences in their practical applications.

The GNN model can be used for most molecular systems “out of the box” with minimal customization but may require hyperparameter tuning to achieve good generalization. Due to its flexibility and expressive power, with the model presented here containing 356,000 parameters, the GNN classifier is somewhat sensitive to overfitting the training data. Again, one could train a smaller GNN model at the empirically observed cost of slower convergence to inferior evaluation minima. Still, the model evaluates relatively quickly with 35 training iterations, each comprising some hundreds of molecules per second on a V100 GPU compute and ∼1 per second on a single CPU. During evaluation, the current performance bottleneck is more often the conversion from MD trajectory output files into the appropriate data format for the GNN model than the model forward pass itself, with 500 trajectory frames of 20 Å^3^ bulk systems taking usually only several minutes to analyze.

The performance of the SF classifier strongly depends on the handcrafted input features. The molecular SFs proposed here do provide the flexibility to capture complex environments in molecular solids but need to be carefully chosen for each new system. This includes both the point-vector representation of the respective molecule and the tunable parameters of the SF.

For larger and more flexible molecules, it might be necessary to expand the molecular SFs to explicitly account for conformational changes, for example, by introducing SFs that depend on different vectors in the same molecule, e.g., corresponding to rigid fragments within the molecule. As with any handcrafted descriptor, this requires a certain level of insight and intuition about the system to be studied. Furthermore, it is desirable to keep the number of molecular SFs small since calculating the input descriptors is the main computational cost when evaluating the SF classifier; that is, a careful selection of new SFs is crucial. The GNN classifier is more general and can be straightforwardly upgraded with more sophisticated geometric features, convolutional methods, or global aggregators to capture longer-range intra- and intermolecular dependencies efficiently within a given system. Today, such architectural improvements are relatively well understood and adoptable “off the shelf”.

Both models are trivially parallelizable as they only require information about a given molecule and its environment and are reasonably computationally cheap for postprocessing molecular simulation data. For on-the-fly usage of local environment information, for example, in enhanced sampling, both methods introduce a computational overhead compared to standard MD. The evaluation of the GNN classifier will generally be slower than the evaluation of a simple empirical force field due to the added computational complexity and, correspondingly, will increase the computational cost. The added cost of the SF classifier is dominated by the choice and number of descriptors, while evaluating the simple classification MLP is negligible. For smaller and simpler molecules, the SF classifier is, therefore, computationally more efficient than the GNN. For larger and more complex molecules, the situation might reverse as the set of required molecular SFs becomes larger and more complex. In simulations employing more accurate and costly interaction potentials, e.g., machine learning potentials, the evaluation of either classifier will only marginally contribute to the overall computational cost.

Our tools are also applicable to multicomponent systems, such as cocrystals, and can be used to identify defects, such as impurities, vacancies, surfaces, or interfaces. The main challenge in these more complex scenarios is the preparation of labeled training data for the supervised learning task.

The two classification models presented in this study provide a general approach for the analysis and interpretation of simulation data in molecular solids. This will be particularly useful for the study of structural transformations, including nucleation and growth. Additionally, information about the local environment can be used to construct collective variables used in enhanced sampling of structural transformations, as we have shown previously for atomistic systems.^19,20^ We expect that the characterization of local structural motifs using classification models will become an essential tool in the simulation of molecular solids as these models are easy to train and extremely versatile.

## Supporting Information Available

Details of descriptors, architecture, and training of the machine learning models and of the MD simulations used to create training and test data and molecular simulation examples (PDF)
