# Supplementary material for: Machine Learning Classification of Local Environments in Molecular Crystals
Source: J Chem Theory Comput. 2024 Jul 3;20(14):6197–206. doi: 10.1021/acs.jctc.4c00418 (PMC11270820; doi:10.1021/acs.jctc.4c00418)
Supplement: Supplementary file 1 — ct4c00418_si_001.pdf [file ct4c00418_si_001.pdf]

Supplementary Information
Machine learning classification of local environments in molecular crystals

Daisuke Kuroshima,[1] Michael Kilgour,[1] Mark E. Tuckerman,[1,2,3,4] and Jutta Rogal[1,5,*]

[1] *Department of Chemistry, New York University (NYU), New York, New York 10003, USA.*
[2] *Courant Institute of Mathematical Sciences, New York University, New York, New York 10012, USA.*
[3] *NYU-ECNU Center for Computational Chemistry at NYU Shanghai, 3663 Zhongshan Rd. North, Shanghai 200062, China.*
[4] *Simons Center for Computational Physical Chemistry at New York University, New York, New York 10003, USA.*
[5] *Fachbereich Physik, Freie Universität Berlin, 14195 Berlin, Germany.*

I. CRYSTAL STRUCTURES OF UREA AND NICOTINAMIDE POLYMORPHS

Figs. S1 and S2 visualize the different polymorphs of urea and nicotinamide, respectively. These structures have been visualized using Ovito [1].

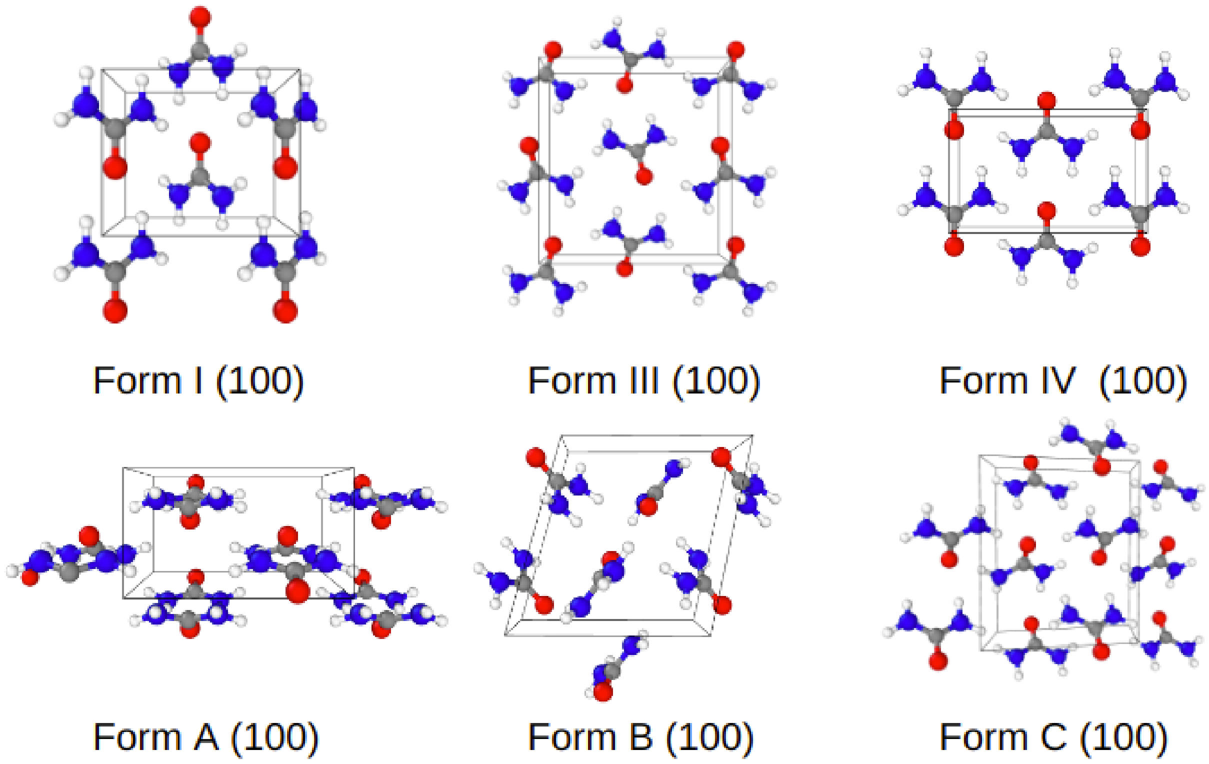

FIG. S1: Crystal structures of six urea polymorphs used in this study viewed along the [100] direction, including the experimentally crystallized forms I, III, and IV, as well as computationally proposed forms A, B, and C. [2–5]

*Electronic address: jutta.rogal@nyu.edu

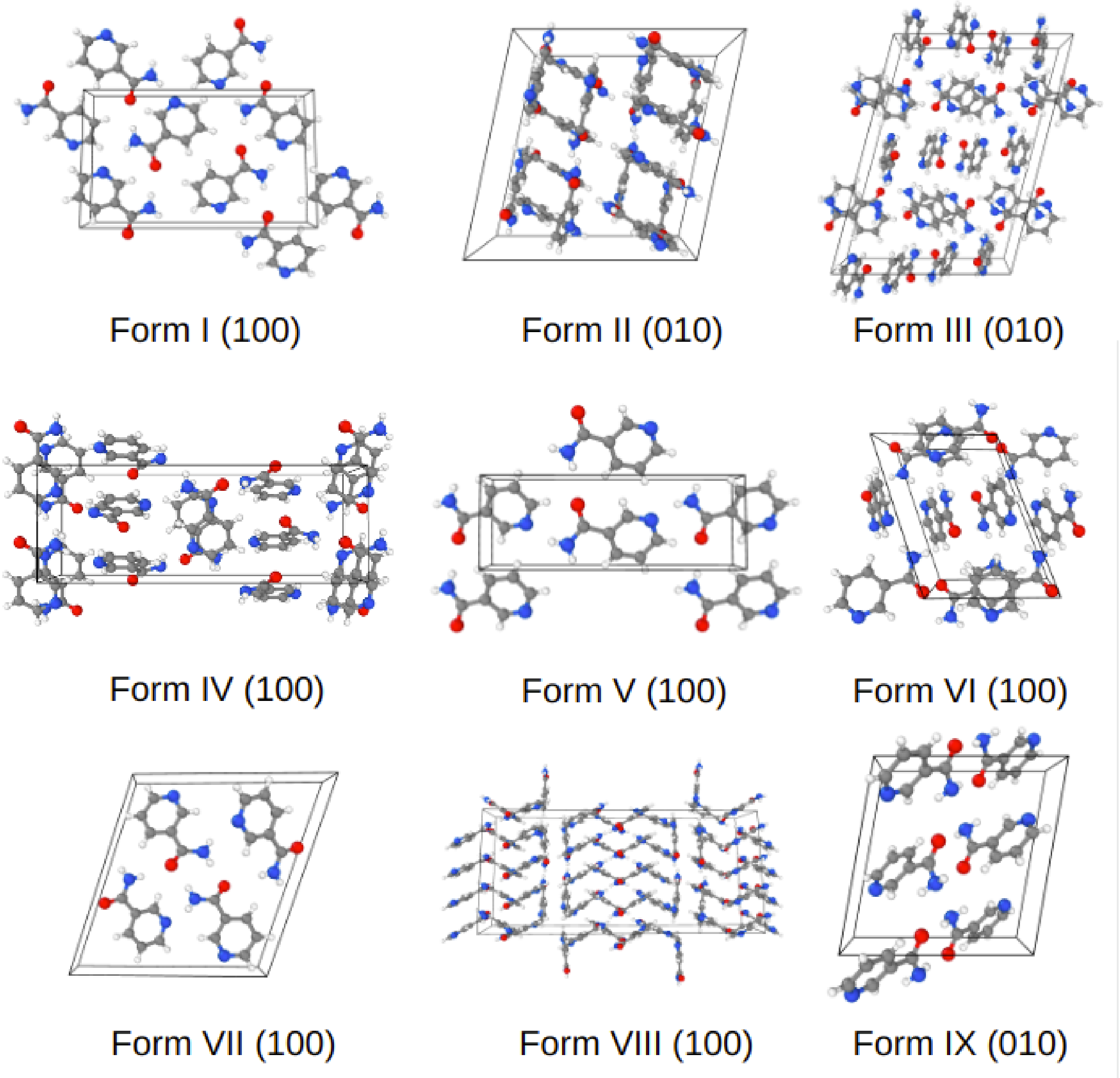

FIG. S2: Crystal structure of nine nicotinamide polymorphs used in this study. Form I, IV, V, VII, and VIII are viewed along the [100] direction, and Form II, III, VI, and IX along [010].

II. MOLECULAR SYMMETRY FUNCTIONS AND TRAINING

The cutoff function used in the molecular symmetry functions has the following form [6]

$$f_c(\mathbf{r}_{IJ}) = \begin{cases} 1 & \text{if } |\mathbf{r}_{IJ}| < r_{\text{min}} \\ \frac{1}{2}\left(\cos\left[\frac{(|\mathbf{r}_{IJ}|-r_{min})}{r_c-r_{min}}\pi\right]+1\right) & \text{if } r_{\text{min}} < |\mathbf{r}_{IJ}| \leq r_c \\ 0 & \text{if } |\mathbf{r}_{IJ}| > r_c \end{cases} \tag{1}$$

where $|\mathbf{r}_{IJ}|$ is the distance between molecule I and J. The cutoff radii are set to $r_{\text{min}} = 9.8$ Å and $r_c = 10.0$ Å for urea and $r_{\text{min}} = 6.8$ Å and $r_c = 7.0$ Å for nicotinamide. A set of input functions was carefully selected by computing the distributions of symmetry function values for a series of the tunable parameters R_s, $\cos\theta_S$, η, and κ. The overlap of distributions for different polymorphs were compared and parameters resulting in small overlaps were selected. In total, 24 molecular symmetry functions were selected for both urea and nicotinamide. The corresponding values for the parameters are given in Tab. S1 for urea and Tab. S2 for nicotinamide.

TABLE S1: Parameters of the molecular symmetry functions used for urea.

symmetry function	R_s	$\cos\theta_S$	η	κ	vector
S_1^I					
1	6.16	-	2.44	-	-
2	6.28	-	2.68	-	-
3	6.76	-	1.00	-	-
4	6.88	-	1.00	-	-
S_2^I					
5	-	-	-	2.50	-
6	-	-	-	4.54	-
7	-	-	-	4.90	-
8	-	-	-	6.22	-
S_3^I					
9	-	0.368	1.00	-	C-O
10	-	0.08	1.00	-	C-O
11	-	0.36	1.12	-	C-O
12	-	0.28	6.76	-	C-O
13	-	-0.64	3.28	-	N-N
14	-	-0.36	3.28	-	N-N
15	-	0.88	3.28	-	N-N
16	-	1.00	3.28	-	N-N
S_4^I					
17	-	-	-	2.50	C-O
18	-	-	-	3.58	C-O
19	-	-	-	4.78	C-O
20	-	-	-	8.26	C-O
21	-	-	-	2.50	N-N
22	-	-	-	8.12	N-N
23	-	-	-	8.24	N-N
24	-	-	-	8.36	N-N

The parameters for symmetry functions were adjusted by comparing the histograms of symmetry functions with different parameters. The overlap of the histograms was calculated for each polymorph, and eight parameters were selected for each of the three types of symmetry functions (one depending on molecule distances and two depending on two different molecule vectors). These symmetry functions were applied to trajectory of each bulk system, and the resulting calculations from each molecule at each snapshot were stored for use in the classification NN.

To train the classification NN with these sets of descriptors, 5,000 and 10,000 training samples were used for urea and nicotinamide, respectively.

TABLE S2: Parameters of the molecular symmetry functions used for nicotinamide.

symmetry function	R_s	$\cos\theta_S$	η	κ	vector
S_1^I					
1	3.75	-	1.26	-	-
2	5.25	-	0.01	-	-
3	4.9	-	0.1	-	-
4	5.9	-	0.016	-	-
S_2^I					
5	-	-	-	1.06	-
6	-	-	-	0.51	-
7	-	-	-	1.03	-
8	-	-	-	2.41	-
S_3^I					
9	-	0.01	0.66	-	C-C
10	-	1.66	0.01	-	C-C
11	-	2.9	0.9	-	C-C
12	-	1.69	4.0	-	C-C
13	-	0.56	3.0	-	O-N
14	-	0.66	0.01	-	O-N
15	-	1.18	17.2	-	O-N
16	-	1.15	2.2	-	O-N
S_4^I					
17	-	-	-	0.13	C-C
18	-	-	-	0.33	C-C
19	-	-	-	5.05	C-C
20	-	-	-	3.2	C-C
21	-	-	-	1.05	O-N
22	-	-	-	0.57	O-N
23	-	-	-	2.36	O-N
24	-	-	-	13.22	O-N

III. GRAPH MODEL HYPERPARAMETERS AND TRAINING

The graph neural network classifier was constructed with one convolutional layer, a nodewise fully-connected layer, followed by two fully-connected layers after graph aggregation. The graph convolution cutoff was 6 Å. The feature depth was 256 throughout, except for during message passing where it was bottlenecked down to 128. Regularization was added with a dropout probability of 0.5 on all fully-connected layers, graphwise layernorm on the graph nodes, and standard layernorm on the graph embedding. We used the Adam optimizer [7] with a constant learning rate of 10^{-4}, and a batch size of 5, synthesized via gradient accumulation over 5 MD snapshots.

The train and test datasets were comprised of 1050 and 250 MD snapshots, respectively, sampled at randomly spaced time intervals, containing on average 370 molecules each, adding up to approximately 390k total molecular environments. Convergence studies showed similar convergence on as little as 10% of this data, which is unsurprising, since at low temperature, most local molecular environments for a given polymorph should be very similar.

IV. DATASET PREPARATION

Bulk periodic molecular dynamics trajectories of the known polymorphs of urea and nicotinamide were undertaken under the following conditions. Simulations were undertaken using the LAMMPS [8] molecular dynamics program. Simulations were run for 1 ns with a time step $\Delta t = 1$ fs in the NPT ensemble using a Nosé-Hoover thermostat and barostat implemented in LAMMPS [9–12].

In this work, we employed the AMBER force field for urea and nicotinamide which relies on second generation of Generalized Amber Force Field (gaff2) [13]. Partial charges for urea were taken from OPLS [14] and for nicotinamide using RESP-charges from PBE calculations [15].

Simulation box sizes were set as the minimum number of unit cell replicas in each cell direction to achieve at least the desired box length, where box lengths of 20 Å and 40 Å were used. The 20 Å samples were used in the GNN model for training on periodic bulk structures. The 40 Å boxes were used to carve out spheres with a 30 Å diameter to create molecular environments on a surface. Surface molecules were identified as having intermolecular coordination numbers less than 20, with that value identified via coordination number histograms within several test clusters, and visually confirmed by inspection of the clusters themselves. The initial configurations of nicotinamide gas phase clusters used were generated in the same way as those of urea crystal and melt, and placed in large periodic boxes to simulate vacuum.

Trajectories were run at temperatures of 100 K and 200 K for urea crystal polymorphs, and 350 K for the supercooled melt, and at 100 K and 350 K for nicotinamide crystal polymorphs and 350 K for the supercooled melt. These temperatures were chosen to ensure that sample structures remained crystalline for each polymorph, while supercooled melts remained liquid.

Melt structures were prepared starting from a stable crystal. After relaxing the system, we gradually increased the temperature from 350 K to 2,000 K over a duration of 10 picoseconds to melt the system. Subsequently, we reduced the temperature of the system back to 350 K on the same timescale. A simulation was then run for 1 nanosecond, and the resulting data was used to characterize the molten structure.

The interface structure was prepared using form I and IV urea structures. To avoid having two moving interfaces, we fixed one of the interface of form I and IV in the z-dimensions, then proceeded to relax the system using MD simulation.

The collected data were randomly divided into testing and training sets. Various sizes of training data, ranging from N = 100 to N = 50,000 unique molecular environments, were used.

V. SYMMETRY FUNCTION CLASSIFIER ACCURACY

Figs. S3-S4 show the evaluation accuracy of the SF classifier on high temperature samples of urea and nicotinamide, respectively. The overall accuracy is nearly perfect in both cases. Note that the SF classifier was only trained on bulk samples, therefore surface vs. bulk classification performance is omitted in this analysis.

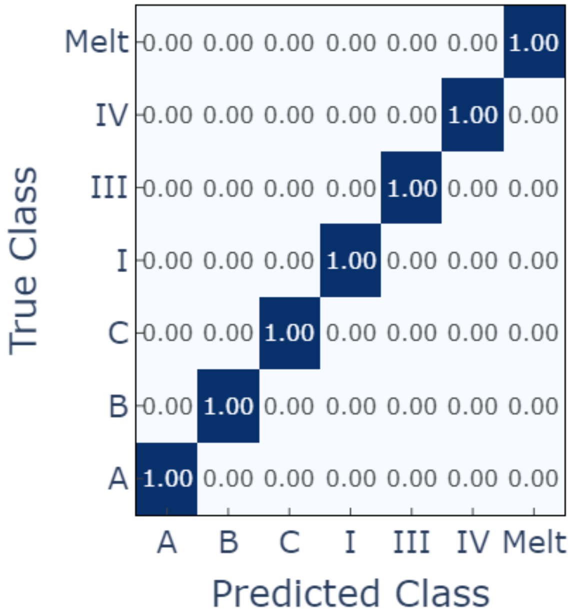

FIG. S3: Confusion matrix for the symmetry function classifier on the polymorphs of urea at 200 K for crystals and 350 K for melt. Micro F1 score=1.0.

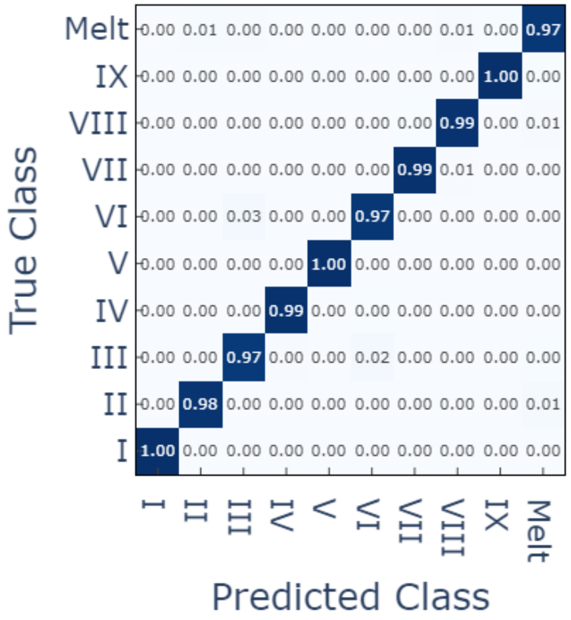

FIG. S4: Confusion matrix for the symmetry function classifier on the polymorphs of nicotinamide at 350 K. Micro F1 score=0.986.

[1] A. Stukowski, Model. Simul. Mater. Sci. Eng. **18**, 015012 (2009).
[2] S. Swaminathan, B. Craven, and R. McMullan, Acta Crystallogr. B. **40**, 300 (1984).
[3] A. Olejniczak, K. Ostrowska, and A. Katrusiak, J. Phys. Chem. C **113**, 15761 (2009).
[4] F. Giberti, M. Salvalaglio, M. Mazzotti, and M. Parrinello, Chem. Eng. Sci. **121**, 51 (2015).
[5] C. Shang, X.-J. Zhang, and Z.-P. Liu, Phys. Chem. Chem. Phys. **19**, 32125 (2017).
[6] M. Chen, M. A. Cuendet, and M. E. Tuckerman, J. Phys. Chem. B **137**, 024102 (2012).
[7] D. P. Kingma and J. Ba, CoRR **abs/1412.6980** (2014).
[8] A. P. Thompson, H. M. Aktulga, R. Berger, D. S. Bolintineanu, W. M. Brown, P. S. Crozier, P. J. in 't Veld, A. Kohlmeyer, S. G. Moore, T. D. Nguyen, et al., Comp. Phys. Comm. **271**, 108171 (2022).
[9] W. Shinoda, M. Shiga, and M. Mikami, Phys. Rev. B **69**, 134103 (2004).
[10] G. J. Martyna, D. J. Tobias, and M. L. Klein, J. Chem. Phys. **101**, 4177 (1994).
[11] M. Parrinello and A. Rahman, J. Appl. Phys. **52**, 7182 (1981), ISSN 0021-8979.
[12] M. E. Tuckerman, J. Alejandre, R. López-Rendón, A. L. Jochim, and G. J. Martyna, J. Phys. A: Math. Gen. **39**, 5629 (2006).
[13] X. He, V. H. Man, W. Yang, T.-S. Lee, and J. Wang, J. Chem. Phys. **153** (2020).
[14] E. M. Duffy, D. L. Severance, and W. L. Jorgensen, Isr. J. Chem. **33**, 323 (1993).
[15] J. Wang, P. Cieplak, and P. A. Kollman, J. Comput. Chem. **21**, 1049 (2000).
